# Application Value of Combined Diagnosis of Ultrasound, MRI, and X-Ray in Developmental Dysplasia of the Hip in Children

**DOI:** 10.1155/2022/1632590

**Published:** 2022-01-19

**Authors:** Jian Li, Bo Zhao, Honghua Ji, Wei Ding

**Affiliations:** ^1^Department of Ultrasonography, Binzhou Hospital of Traditional Chinese Medicine, Binzhou 256601, Shandong, China; ^2^Department of Ultrasonography, People's Hospital of Rizhao, Rizhao 276826, Shandong, China

## Abstract

**Objective:**

To explore the application value of the combined diagnosis of ultrasound, MRI, and X-ray in developmental dysplasia of the hip (DDH) in children.

**Methods:**

Ninety children with suspected DDH admitted to our hospital from June 2017 to June 2020 were selected as the research objects to conduct a retrospective study. According to the age of the children, they were divided into a group with 0–6 months (group X), a group with 7–12 months (group Y), and a group older than 12 months (group Z), with 30 cases in each group. X-ray and high-frequency ultrasound were performed in all groups, and MRI examination was added to the children in groups Y and *Z* to compare the diagnostic value of the three imaging examinations in DDH children.

**Results:**

No obvious differences in the general data and maternal risk factors were observed among the three groups (*P* < 0.05). The final comprehensive diagnostic results were taken as the gold standard, including 23 cases with acetabular dysplasia, 28 cases with subluxation of the femoral head, 31 cases with complete dislocation of the femoral head, and 8 non-DDH cases. The diagnostic accuracy of the three methods from high to low was MRI, high-frequency ultrasound, and X-ray, with obviously higher diagnostic accuracy of MRI than that of X-ray (*P* < 0.05). The ROC curves showed that the diagnostic efficacy from high to low was MRI + high-frequency ultrasound + X-ray, high-frequency ultrasound + X-ray, MRI, high-frequency ultrasound, and X-ray.

**Conclusion:**

Ultrasound combined with X-ray has obvious advantages in the diagnosis of children at low months of age, while MRI has outstanding advantages in the diagnosis of children at high months of age. MRI combined with ultrasound and X-ray can significantly improve the diagnostic accuracy of DDH and provide objective data support for the clinical treatment of children.

## 1. Introduction

Developmental dysplasia of the hip (DDH) in children is the most common hip disease in pediatric orthopedics. With complex pathological structure and dynamic development, DDH mainly includes acetabular dysplasia, subluxation of the femoral head, and complete dislocation of the femoral head [1–4]. According to clinical observation, early diagnosis and treatment are crucial for DDH children, and early diagnosed children can achieve satisfactory results under timely conservative treatment. Otherwise, the growth of children will not only increase the difficulty of treatment but also reduce the efficacy. Nowadays, still occupying a large proportion, many DDH children do not receive timely treatment. These children may have diseases such as gait abnormalities and spinal abnormalities and may be prone to long-term and chronic degenerative hip diseases in adulthood, accompanied by long-term pain, or even disability in severe cases [5–8]. It can be seen that early diagnosis is the key to guiding clinical decision-making and obtaining good prognosis, and imaging examination is the main method to determine DDH at present. Among them, ultrasound, X-ray, and MRI are the main methods for clinical diagnosis of DDH in children. There are many studies on the three examination methods applied in the diagnosis of DDH, but no unified and effective imaging diagnosis scheme has been formed. Based on this, this study explored the application value of MRI combined with X-ray and ultrasound in the diagnosis of DDH in children.

## 2. Study Protocol

### 2.1. Case Screening

The inclusion criteria for children were formed as follows according to research objectives and procedures. (1) Children were initially diagnosed as suspected DDH cases according to routine physical examination; (2) children had one or more of the following signs and symptoms: (a) asymmetric hip or thigh patterns, (b) unilateral hip joint retraction or bilateral dislocation and widened perineum, (c) positive Allis sign, (d) positive result in left abduction test, (e) positive Ortolani sign, (f) positive Barlow sign, (g) less limb movement and weak pedaling in children, and (h) the hip joint had clicking; (3) children were no more than 12 years old; (4) children had complete medical records; (5) the family members of the children were informed of and agreed to this study; and (6) children received the imaging examinations for the first time.

#### 2.1.1. Exclusion Criteria

(1) Children had malformed and developmental dislocation of the hip; (2) before enrollment, children did not receive the imaging examinations required for this study; (3) children had secondary joint dislocation; and (4) children had multiple joint contracture or cerebral palsy.

According to the above criteria, 90 children with suspected DDH admitted to our hospital from June 2017 to June 2020 were selected for a retrospective study.

### 2.2. Grouping

According to their age, the 90 enrolled children were divided into a group with 0–6 months (group X), a group with 7–12 months (group Y), and a group older than 12 months (group Z), with 30 cases in each group. The hospital ethics committee approved the study and supervised the implementation of the study protocol.

### 2.3. Methods

X-ray and high-frequency ultrasound were performed in all groups, and MRI examination was added to the children in groups Y and Z. Sedation was needed during MRI examination, so MRI was not suitable for low-month children.

#### 2.3.1. X-Ray Examination

The examiner helped the child lie quietly on his back, with the distance between his lower limbs as wide as his shoulder breadth, and the tiptoes rotated about 20° inward. The Primary Diagnost DR machine (manufacturer: Philips) was used for scanning, and the anteroposterior radiographs of the hip were taken to obtain clear X-ray films. According to the parameters such as acetabular angle, central-edge angle, Perkin square, Calve line, and Shonton line, the acetabular development of the children was determined [9–12].

#### 2.3.2. High-Frequency Ultrasound Examination

The child stayed lateral, with the hip joints buckled to 30°. The examiner used a color Doppler ultrasound diagnosis apparatus (model: DC-N2S) with a linear array probe of 5–7.5 MHz to scan the hip joints of the child and to obtain the coronal plane of femoral greater trochanter. Then, the probe was perpendicular to the scanning plane, and continuous scanning was performed. The coronal plane images were obtained by parallel scanning from the back to the ventral part. The first line (baseline) was drawn from the top of the acetabular cartilage to the lateral line that was tangential to the iliac bone plate. The lower edge of the iliac bone in the acetabular fossa was connected with the external and lower angle of the acetabular bone, as the second line. The midpoint of the labrum articularis was connected with the external and lower angle of the acetabular bone, as the third line. The angle between the first and second lines was *α*, and the angle between the second and third lines was *β* [12–15].

#### 2.3.3. MRI Examination

The selected instrument was 3.0 T superconducting Avanto magnetic resonance instrument (manufacturer: Siemens). Appropriate sedation could be given to the child who could not cooperate with the examiner, and MRI examination was performed after the child was asleep. The child was supine, with both lower limbs straight and the tiptoes buckled. Small soft pads were placed under both ankle joints, and the ankle and knee joints were tied with fixing straps. Abdominal coil scanning was performed to fully cover both bilateral hip joints and the distal femur. Phase scanning was performed according to the order of axial, coronal, and sagittal positions, and then the scanning line was scanned in parallel with the femoral neck axis [16–18]. Scanning with the T_2_TSE sequence was performed to obtain the oblique sagittal image of the femoral neck. The center of the femoral head was connected with the midpoint of the narrowest part of the femoral neck to obtain the femoral neck axis, which formed a *Q* angle with the horizontal line. The distal femur of the affected side was scanned with the T_1_TSE sequence to obtain the axial image. The condyle line was formed by connecting the posterior margin of the epiphyseal cartilage of the internal and external condyles, forming a *B* angle with the horizontal line. The anteversion of femoral neck was calculated according to angles *Q* and *B*.

### 2.4. DDH Diagnostic Criteria

#### 2.4.1. X-Ray Examination

Acetabular dysplasia could be determined with acetabulum angle >30°, center-edge angle <20°, discontinuous Shonton line, discontinuous Calve line, and Perkin square in the outer lower or outer upper quadrant.

#### 2.4.2. High-Frequency Ultrasound

(1) Complete hip dislocation: *α* < 45° and *β* not available. (2) Hip subluxation: 45° < *α* ≤ 50° and *β* > 77°. (3) DDH: 50° < *α* ≤ 55° and 55° ≤ *β* ≤ 77°. (4) Hip instability: 55°<*α* ≤ 60°and 55 ≤ *β* ≤ 77°. (5) Normal hip: *α* > 60°and *β* < 55°.

#### 2.4.3. MRI Examination

DDH was judged according to the age of the children and the anteversion of femoral neck. The normal anteversion of femoral neck was 25°–35°, which decreased by 1° per one-year increase of age, and children beyond this range could be diagnosed with DDH.

### 2.5. Statistical Treatment

The data obtained in this study were processed by software SPSS22.0 and graphed by software GraphPad Prism 7 (GraphPad Software, San Diego, USA). The study data included enumeration data and measurement data, expressed as *n* (%) and ($\bar{x}+s$) and tested by *X*^2^ and *t*-test. The differences were statistically significant at *P* < 0.05.

## 3. Results

### 3.1. General Data

No obvious differences in the general data and maternal risk factors were observed among the three groups (*P* < 0.05) (see Table 1).

### 3.2. Clinical Diagnostic Results

The final comprehensive diagnostic results were taken as the gold standard, including 23 cases with acetabular dysplasia, 28 cases with subluxation of the femoral head, 31 cases with complete dislocation of the femoral head, and 8 non-DDH cases, as presented in Table 2.

### 3.3. Diagnostic Results of X-Ray, Ultrasound, and MRI

**Figure 1:**
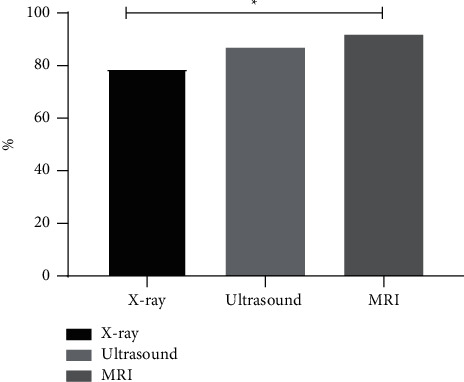
Diagnostic accuracy (%). Note: the abscissa represented the imaging methods, and the ordinate represented the percentage (%). X-ray examination confirmed 71 cases (78.89%) of DDH, ultrasound confirmed 78 cases (86.67%), and MRI confirmed 55 cases (91.67%). ^*∗*^indicated a notable difference in the diagnostic accuracy between X-ray and MRI (*X*^2^ = 4.640, *P* = 0.031).

The diagnostic accuracy of the three methods from high to low was MRI, high-frequency ultrasound, and X-ray, with obviously higher diagnostic accuracy of MRI than that of X-ray (*P* < 0.05) (see Figure 1).

### 3.4. ROC Curves

**Figure 2:**
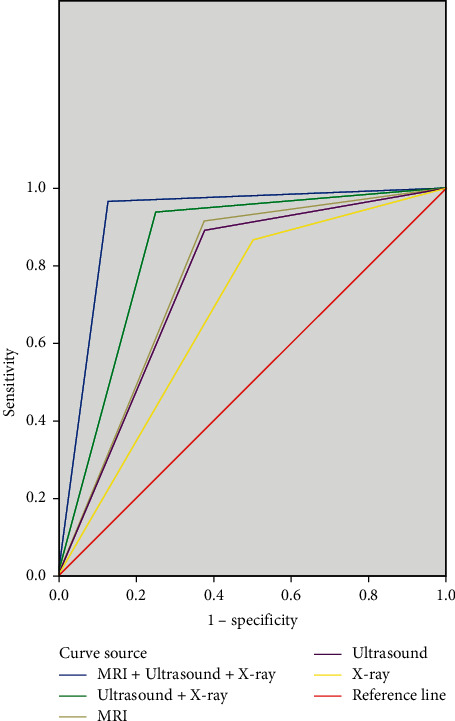
ROC curves.

The ROC curves showed that the diagnostic efficacy from high to low was MRI + high-frequency ultrasound + X-ray, high-frequency ultrasound + X-ray, MRI, high-frequency ultrasound, and X-ray, as shown in Figure 2. The area under the curves is shown in Table 3.

## 4. Discussion

Imaging examination, an important basis for early diagnosis, treatment, and follow-up after intervention of DDH, can directly reflect the hip structure and development of children [19–22]. Since DDH mostly occurs in infants and young children, the subjects for examination are special. In addition, early diagnosis, early treatment, and timely adjustment of the treatment plans play a vital role in the later growth of the children. Therefore, the clinical diagnosis of DDH is inseparable from the support of imaging data from high-frequency ultrasound, X-ray, and MRI. Though there are many related comparative studies at present, these three methods are highly flexible when applied in the diagnosis of infants and young children with DDH, and no complete and effective application plan has been formed. Based on this, this paper explored the application value of the combined diagnosis of ultrasound, MRI, and X-ray in DDH children and tried to explore a feasible scheme for clinical diagnosis and treatment of DDH. In this study, no obvious differences in the general data and maternal risk factors were observed among the three groups (*P* < 0.05). The general data of children were recorded, and the influencing factors that may lead to DDH in children were analyzed. It was found that children in each group were accompanied by different levels of risk factors, such as high birth weight, multiple pregnancy, and oligohydramnios, which were consistent with the clinical status. Therefore, in early screening of DDH newborns, hospitals should strengthen the awareness of the above risk factors to improve the clinical screening rate of neonatal DDH. The final comprehensive diagnostic results were taken as the gold standard, including 23 cases with acetabular dysplasia, 28 cases with subluxation of the femoral head, 31 cases with complete dislocation of the femoral head, and 8 non-DDH cases. The diagnostic accuracy of the three methods from high to low was MRI, high-frequency ultrasound, and X-ray, with obviously higher diagnostic accuracy of MRI than that of X-ray (*P* < 0.05). These results were in line with the study of Walbron [23], suggesting that MRI, ultrasound, and X-ray had certain advantages in the diagnosis of DDH in children. Firstly, DDH usually occurs in newborns or infants, and children with low months of age have not yet shown ossific hip joints. Therefore, ultrasound shows high superiority in the diagnosis of DDH children with low months, and it can be used as the preferred standard for DDH screening in these children, especially for high-risk children. Alassaf et al. [24] have reported that, for newborns with mild dysplasia but stable hips shortly after birth, treatment is not required, but active ultrasonic monitoring is necessary. Secondly, X-ray examination is fast and economic. However, the ossification center of femoral head has not yet formed in infants within 3 months, and indicators such as Perkin quadrant, center-edge angle, and acetabular angle (Sharp angle) can not be detected. Therefore, X-ray examination is not recommended for children within 3 months, which is more reliable in children of 4–6 months when the ossification center of femoral head appears. With the application of digital radiography (DR) technology, digital X-ray imaging is fast, with high definition, low radiation, and strong post-processing function. Therefore, X-ray can become the preferred auxiliary method for the diagnosis and observation of DDH children of 4–6 months. Finally, compared with ultrasound and X-ray examination, the advantage of MRI is mainly reflected in the higher resolution in displaying the soft tissues, which is difficult to achieve by other examination methods. MRI can observe the pathological changes of soft tissue structure in and around the hip joints and then determine the progression and treatment of children diagnosed by ultrasound and X-ray examination in the early stage and constantly update data for subsequent treatment. In summary, MRI is mainly used to display the relationship between the femoral head and the acetabulum after closed reduction or open reduction and can display both cartilage and joint labrum. At the same time, sedation is required for children in this examination. Therefore, MRI is not suitable for children with low months of age.

The ROC curves showed that the diagnostic efficacy from high to low was the MRI + high-frequency ultrasound + X-ray, high-frequency ultrasound + X-ray, MRI, and X-ray. Therefore, this study believed that most of the hip joints of children at low months are cartilage components, and high-frequency ultrasound can better present the unossified hip joint structure. Early screening of DDH is achieved by observing the main structures of the femoral neck epiphyseal plate, lower edge of the iliac bone, synovial reflex, femoral head, cartilage top, turning point, joint capsule, labrum, and bone top in the ultrasound images, which provides a reliable basis for clinical diagnosis. X-ray examination can be used as a routine auxiliary examination for children over three months, which is conducive to further improving the clinical diagnosis rate of children at low age. MRI is of irreplaceable importance in older DDH children, which is more accurate in judging and evaluating the growth of the acetabulum, the pathological changes of the soft tissue structure in and around the hip joints, and the evolution process. It provides very important reference data for clinical diagnosis and treatment. MRI combined with high-frequency ultrasound and X-ray examination can provide more complete and reliable information for the diagnosis and treatment, thus better guiding clinical treatment, prognosis, and dynamic follow-up. However, this retrospective study lacked the follow-up information of some children. Therefore, the application value of X-ray, high-frequency ultrasound, and MRI in the dynamic follow-up of children with DDH should be noted in the subsequent studies.

In conclusion, ultrasound combined with X-ray has obvious advantages in the diagnosis of children at low months of age, while MRI has outstanding advantages in the diagnosis of children at high months of age. MRI combined with ultrasound and X-ray can significantly improve the diagnostic accuracy of DDH and provide objective data support for the clinical treatment of children.

## Figures and Tables

**Table 1:** Comparison of general data (*n* = 30).

Observation indexes	Group X	Group Y	Group Z	*P*
Children condition				
Gender				<0.05
Male	14 (46.67%)	15 (50%)	13 (43.33%)	
Female	16 (53.33%)	15 (50%)	17 (56.67%)	

Injured sides				
Left side	18 (60%)	17 (56.67%)	17 (56.67%)	<0.05
Right side	9 (30%)	8 (26.67%)	9 (30%)	<0.05
Bilateral sides	3 (10%)	5 (16.67%)	4 (13.33%)	<0.05
Birth weight (kg)	3.23 ± 0.36	3.25 ± 0.37	3.24 ± 0.36	<0.05

Maternal risk factors				
Breech presentation	14 (46.67%)	15 (50%)	13 (43.33%)	<0.05
Multiple pregnancy	8 (26.67%)	8 (26.67%)	9 (30%)	<0.05
Oligohydramnios	16 (53.33%)	18 (60%)	17 (56.67%)	<0.05

**Table 2:** Clinical diagnostic results in three groups (*n* (%)).

Observation indexes	Group X	Group Y	Group Z
Acetabular dysplasia	8 (26.67)	8 (26.67)	7 (23.33)
Subluxation of the femoral head	9 (30)	10 (33.33)	9 (30)
Complete dislocation of the femoral head	10 (33.33)	9 (30)	12 (40)

**Table 3:** Area under the curves.

Test result variables	Area	Standard error^a^	Progressive Sig^b^	Progressive 95% confidence interval
MRI + ultrasound + X-ray	0.919	0.069	0.000	0.000–1.000
Ultrasound + X-ray	0.845	0.095	0.001	0.599–1.000
MRI	0.770	0.106	0.012	0.563–0.977
Ultrasound	0.758	0.105	0.017	0.551–0.964
X-ray	0.659	0.111	0.140	0.441–0.876

a, under nonparametric hypothesis; b, null hypothesis, actual area = 0.5.

## Data Availability

Data to support the findings of this study are available upon reasonable request from the corresponding author.
